# Regulation of Angiogenic Functions by Angiopoietins through Calcium-Dependent Signaling Pathways

**DOI:** 10.1155/2015/965271

**Published:** 2015-06-04

**Authors:** Irene Pafumi, Annarita Favia, Guido Gambara, Francesca Papacci, Elio Ziparo, Fioretta Palombi, Antonio Filippini

**Affiliations:** ^1^Department of Anatomy, Histology, Forensic Medicine and Orthopaedics, Unit of Histology and Medical Embryology, Sapienza University of Rome, 00161 Rome, Italy; ^2^Institute of Vegetative Anatomy, Charité Universitätsmedizin, Neuromuscular Group, 10115 Berlin, Germany

## Abstract

Angiopoietins are vascular factors essential for blood vessel assembly and correct organization and maturation. This study describes a novel calcium-dependent machinery activated through Angiopoietin-1/2-Tie receptor system in HUVECs monolayer. Both cytokines were found to elicit intracellular calcium mobilization. Targeting intracellular Ca^2+^ signaling, antagonizing IP_3_ with 2-APB or cADPR with 8Br-cADPR, was found to modulate *in vitro* angiogenic responses to Angiopoietins in a specific way. 2-APB and 8Br-cADPR impaired the phosphorylation of AKT and FAK induced by Ang-1 and Ang-2. On the other hand, phosphorylation of ERK1/2 and p38, as well as cell proliferation, was not affected by either inhibitor. The ability of ECs to migrate following Angs stimulation, evaluated by “scratch assay,” was reduced by either 2-APB or 8Br-cADPR following Ang-2 stimulation and only slightly affected by 2-APB in cells stimulated with Ang-1. These results identify a novel calcium-dependent machinery involved in the complex interplay regulating angiogenic processes showing that IP_3_- and cADPR-induced Ca^2+^ release specifically regulates distinct Angs-mediated angiogenic steps.

## 1. Introduction

Angiogenesis is a complex remodeling process characterized by the sprouting of new blood vessels from preexisting ones, occurring mainly during embryonic development and in pathological processes such as cancer. During the early stages of this process, endothelial cells (ECs) are stimulated to form new capillaries by growth factors such as vascular endothelial growth factor (VEGF) and fibroblast growth factor (FGF). Angiopoietins (Angs) play a fundamental role in the subsequent maturation step, in which microvessels acquire a layer of mural cells, pericytes, and smooth muscle cells, which are critical for the development and maintenance of functional vasculature [1, 2]. Experimental evidence identifies the Angs and their Tie receptors as important regulators of tumor-induced angiogenesis and metastasis [3, 4]. So far, a major effort in the field of angiogenesis control has centered on the VEGF-VEGF receptor system [5, 6]. However, despite partial success, resistance to anti-VEGF therapy, resulting from a variety of mechanisms, remains a major obstacle [7–9]. The search for novel key downstream effectors as a possible common “signaling hub” between Angs and other known growth factors is therefore of potential significance in the perspective of angiogenesis control in cancer.

The Tie receptors and their Angiopoietin (Ang) ligands have been identified as the second vascular tissue-specific tyrosine kinase receptor system. Angs-Tie system is essential during embryonic vessel assembly and for the correct organization and maturation of newly formed vessels and functions as a key regulator of adult vascular homeostasis in the later stages of the angiogenic cascade. Thus, whereas VEGF signals promote initiating events in angiogenesis, such as ECs sprouting and proliferation, Ang-Tie signals appear to promote ECs survival and vascular assembly, stability, and maturation [10]. The best-characterized Angs are Angiopoietin-1 (Ang-1) and Angiopoietin-2 (Ang-2), which are secreted glycoproteins with a dimeric structure and molecular weight of approximately 75 kDa [11] showing about 60% amino acid sequence homology. Ang-1 is expressed by smooth muscle cells and other perivascular cells and acts in a paracrine manner as agonist of the endothelial Tie-2 receptor, whereas Ang-2 is considered as its antagonist although also reported to context-dependently act as a Tie-2 agonist inducing receptor phosphorylation [12–14]. The molecular basis for agonistic versus antagonistic functions of Ang-2 has not been unraveled. Cell type specific effects, the degree of endothelial confluence, the duration of Ang-2 stimulation, concentration dependent effects, and the presence of the coreceptors such as Tie-1 have all been implicated in controlling agonistic versus antagonistic function of Ang-2 [15–18]. Ang-1 induces tyrosine phosphorylation of Tie-2 in ECs and activates downstream signaling pathways such as mitogen-activated protein kinase (MAPK) pathways. In adult tissues, Ang-1 does not induce endothelial chemotaxis or proliferation but exerts a dual role: it stimulates angiogenesis at sites of vascular remodeling contributing to the formation of capillary sprouts, while it stimulates prosurvival pathways and promotes vascular quiescence in mature vessels, preventing apoptosis and inflammation through the activation of PI3K-AKT and MAPK/ERK signaling pathways [19–21]. Ang-2 is almost exclusively expressed by ECs where it is stored in Weibel-Palade bodies (WPB) [22, 23]. Following cytokine activation of the endothelium, Ang-2 is rapidly released and acts in an autocrine manner on Tie-2 receptor binding as homodimers or multimers. Under physiological conditions, in the adult tissues Ang-2 is expressed in regions of vascular remodelling, during vascularization of the retina or during vessel formation/regression of ovarian corpus luteum. Ang-2 expression is also upregulated under pathological conditions, for example, in the endothelium of tumors [24–26]. Interestingly, experimental evidence indicates that signaling downstream of Tie-2 activation is influenced by the subcellular localization of the receptor, which is different in confluent versus sparse ECs [1, 27]. In these studies, Ang-1 produced stronger AKT signaling in confluent cells, in which Tie-2 was localized at cell/cell junctions, and stronger ERK activation when cells were sparse and Tie-2 was localized at cell/matrix junction, indicating that the effect of Ang-1 on Tie-2 activation may depend on the nature of cell/cell or cell/matrix contacts. Less well known is the role of calcium (Ca^2+^) in the regulation of angiogenic processes. Ca^2+^ is a crucial point of intersection for many distinct molecular signaling pathways that promote and modulate angiogenesis. Specific Ca^2+^ signatures rely upon spatiotemporal variations in [Ca^2+^]_i_ [28] and are mainly based on three second messengers, namely, inositol trisphosphate (IP_3_) and cyclic adenosine diphosphoribose (cADPR), which mobilize Ca^2+^ from endoplasmic reticulum (ER) stores, and nicotinic acid adenine-dinucleotide phosphate (NAADP), which triggers Ca^2+^ release from acidic organelles [29, 30]. We have previously demonstrated that histamine H1 receptors mediate NAADP-dependent Ca^2+^ signaling in ECs [31] and have recently identified that in HUVECs (human umbilical vein endothelial cells) VEGF increases [Ca^2+^]_i_ by mobilizing Ca^2+^ from internal stores. The latter study demonstrated the direct role of NAADP in VEGF-induced Ca^2+^ mobilization from acidic organelles and its important involvement in the control of angiogenic processes [32]. To our knowledge, the role of Ca^2+^ signaling in the regulation of Ang-1/Ang-2-induced angiogenesis has not been investigated yet. In the present work, we identify a novel pathway for Angs-Tie signaling whereby receptor activation leads to IP_3_- and cADPR-dependent Ca^2+^ release and show that each of these Ca^2+^ mobilizing second messengers specifically controls distinct Angs-mediated angiogenic steps.

## 2. Materials and Methods

### 2.1. Cell Culture

Human umbilical vein endothelial cells (HUVECs) were obtained from Lonza Sales Ltd., cultured in EGM-2 Endothelial Cell Growth Medium-2 (Endothelial Basal Medium EBM-2 + EGM-2 Bullet Kit, Lonza), + 100 mM penicillin/streptomycin (Sigma). Cells were maintained at 37°C in a humified 5% (vol/vol) incubator and grown to reach the confluence. Monolayers were used at passages 1 to 6.

### 2.2. Calcium Imaging

Cells were incubated in EGM-2 containing 3.5 *μ*M Fura-2-AM (Invitrogen) for 1 h at 37°C and then rinsed with Hanks' Balanced Salt Solution (HBSS Sigma) or Krebs-Henseleit-HEPES (KHH) buffer (140 mM Na^+^, 5.3 mM K^+^, 132.4 mM Cl^−^, 0.98 mM PO_4_^2−^, 1.25 mM Ca^2+^, 0.81 mM Mg^2+^, 5.5 mM glucose, and 20 mM HEPES). Plates were placed into a culture chamber kept at 37°C controlled temperature on the stage of an inverted microfluorimeter (Nikon TE2000E) connected to a cooled CCD camera (512B Cascade, Princeton Instruments). Samples were illuminated alternately at 340 and 380 nm using a random access monochromator (Photon Technology International) and emission was detected using a 510 nm emission filter. Images were acquired (1 image/sec ratio) using MetaFluor software (Universal Imaging Corporation). Calibration of the signal was obtained at the end of each experiment by maximally increasing intracellular Ca^2+^-dependent Fura-2-AM fluorescence with 5 *μ*M ionomycin (ionomycin calcium salt from* Streptomyces conglobatus*, Sigma) followed by recording minimal fluorescence in Ca^2+^-free medium. [Ca^2+^]_i_ was calculated according to previously described formulas [33].

### 2.3. Western Blot

Cells were first starved in EBM-2 for 4 h and then incubated with BAPTA-AM (Sigma, 20 *μ*M), 2-APB (Sigma 75 *μ*M), or 8Br-cADPR (Sigma 30 *μ*M) for 30 min before stimulation for 20 min with 100 ng/mL Angiopoietin-1 or 200 ng/mL Angiopoietin-2 (PeproTech). Cells were washed with cold PBS before adding lysis buffer 10X (Cell Signaling), PI, and PMSF (Sigma 1 mM). After determining protein concentration by a BCA kit (Thermo Scientific), 25 *μ*g–35 *μ*g protein of each sample was loaded on 8%–10% SDS-PAGE. The proteins were subsequently blotted onto a nitrocellulose membrane and the membrane treated with a blocking solution TTBS + 5% milk (ECL prime blocking agent, GE Healthcare). The following primary antibodies were used: Phospho-p42/44 MAPK (T202/Y204: E10, Cell Signaling, 1 : 1,000), Phospho-AKT (Ser-473, Cell Signaling, 1 : 1,000), Phospho-FAK (Tyr925, Cell Signaling, 1 : 1,000), Phospho-p38 MAP Kinase (Thr180/Tyr182, Cell Signaling, 1 : 1,000), AKT (Cell Signaling, 1 : 1,000), ERK 2 (Santa Cruz, 1 : 1,000), p38 (Santa Cruz, 1 : 1,000), and FAK (Cell Signaling, 1 : 1,000). All the antibodies were diluted in TTBS 5% BSA. After three washes in TTBS, the membrane was incubated with a secondary HRP-conjugated stabilized goat anti-mouse (Pierce, 1 : 10,000) and stabilized peroxidase-conjugated polyclonal goat anti-rabbit (Bio-Rad, 1 : 10,000) 1 h at RT. To ensure equal loading membranes were reprobed with monoclonal HRP-conjugated anti-*β*-actin (Sigma). The intensity of Western blot bands was quantified by Image J software from at least three independent experiments, normalized to both total amount of the protein indicated and *β*-actin content, and compared with control (vehicle) set as 1.

### 2.4. Scratch Assay

Confluent ECs monolayers plated in 35 mm dishes were scraped along a straight line with a p10 pipette tip to create a narrow gap (scratch). The scratch created was of similar size in the different experimental conditions to minimize any possible variation caused by differences in the width. Debris were removed by a wash with PBS prior to incubation in fresh medium containing Angs in the presence or absence of inhibitors. To record cell migration, images of the wound were acquired at time zero and again 24 h later in an inverted microscope (Nikon Eclipse TS100) equipped with a digital camera (Nikon Ds Fi2, Nis elements F 4.00.00 software). Five random microscopic fields along each wound were photographed.

### 2.5. *In Vitro* Matrigel Assay

Capillary-like endothelial tube formation was evaluated by an angiogenesis* in vitro* angiogenesis assay. 130 *μ*L Matrigel basement membrane matrix growth factor reduced (BD Biosciences) was added to each well of precooled 24-well tissue culture plates. Pipette tips and Matrigel solution were kept cold during the procedure to avoid solidification. The plates were incubated for 1 h at 37°C to allow matrix solution to solidify. 4 × 10^4^ cells in a final volume of 500 *μ*L EBM-2 were seeded onto the surface of each well containing the polymerized matrix. Cells were pretreated with the pharmacological inhibitors indicated or with vehicle alone and stimulated with the specific agonists 100 ng/mL Ang-1 or 200 ng/mL Ang-2 for 4-5 hours at 37°C. Tube formation was inspected under an inverted microscope (Nikon Eclipse TS100) at 20x magnification and images were acquired by a digital camera (Nikon Ds Fi2, Nis elements F 4.00.00 software). The closed polygons formed in five random microscopic fields per well were counted and values averaged.

### 2.6. Statistical Analysis

**Figure 1:**
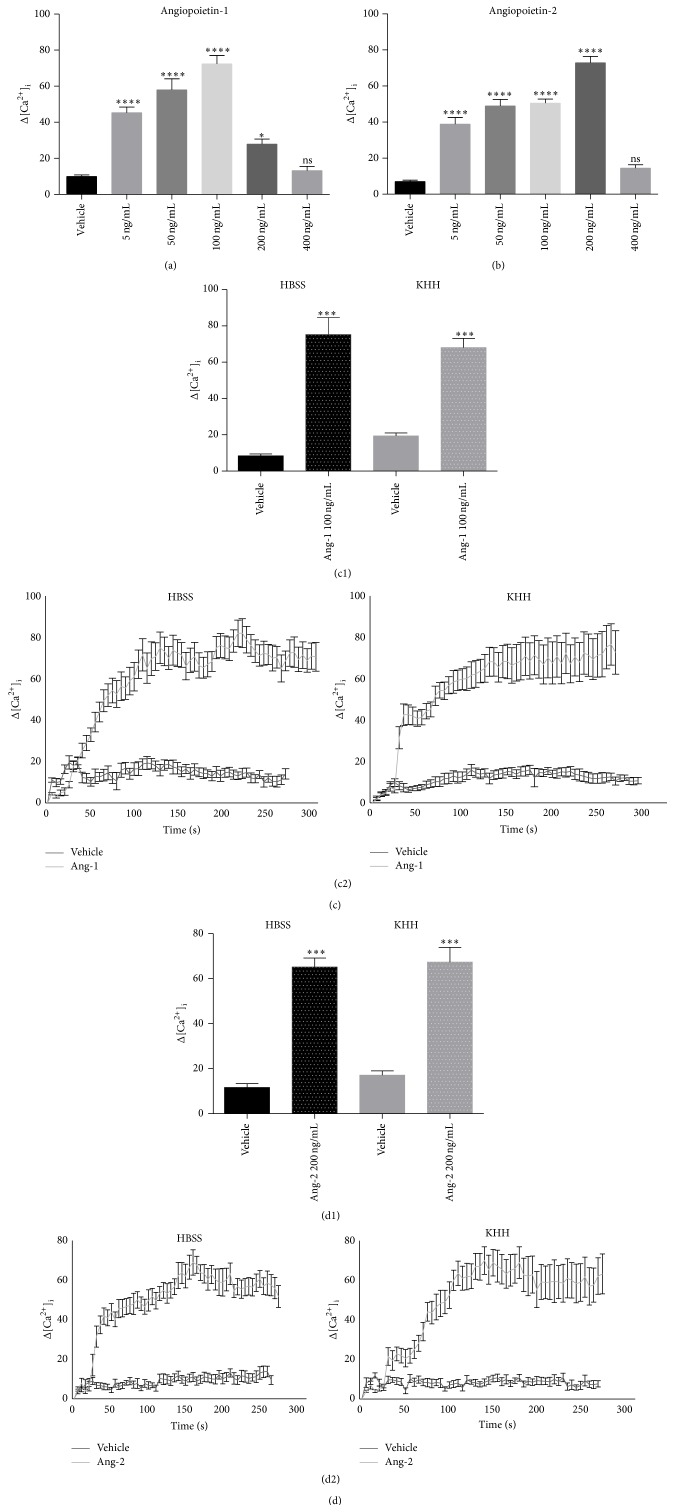
[Ca^2+^]_i_ is increased by Ang-1 and Ang-2 stimulation. Live imaging in Fura-2-AM-loaded single cells. [Ca^2+^]_i_ increases (nM) following stimulation with either Ang-1 or Ang-2 at different concentrations (5 to 400 ng/mL) in Ca^2+^ containing HBSS buffer. Identification of 100 ng/mL Ang-1 and 200 ng/mL Ang-2 as agonist concentrations is most effective in increasing [Ca^2+^]_i_ levels (a, b). Data in bar charts represent mean ± s.e.m. from three independent experiments. Statistical analysis of the data was performed using one-way ANOVA test. ^****^ *P* value < 0.0001, ^*^ *P* value < 0.05. Lack of contribution from Ca^2+^ influx, tested in absence or presence of extracellular calcium (resp., in KHH or HBSS buffer) (c, d). Changes in [Ca^2+^]_i_ levels (nM) are shown as maximum concentration in bar charts (c1, d1) and as representative traces (c2, d2) in cells stimulated with Ang-1 or Ang-2, at the respective maximal concentrations, or with vehicle alone. Agonist-stimulated [Ca^2+^]_i_ increases are substantially the same in the presence or absence of extracellular Ca^2+^. Data in bar charts represent mean ± s.e.m. from three independent experiments. Cell number = 78–155. ^***^ *P* < 0.001 versus vehicle by Student's *t*-test. [Ca^2+^]_i_ values in representative traces are expressed as increase with respect to basal Ca^2+^ concentrations (Δ).

Data are presented as the mean ± s.e.m. resulting from at least three independent experiments. Student's *t*-test was used for statistical comparison between means where applicable. Consider ^*^ *P* < 0.05, ^**^ *P* < 0.01, and ^***^ *P* < 0.001. Statistical analysis of the data in Figures 1(a) and 1(b) was performed using the one-way ANOVA test. Consider ^****^ *P* value < 0.0001, ^*^ *P* value < 0.05. Bands on Western blots were quantified by densitometric scanning of films from three or more independent experiments.

## 3. Results and Discussion

### 3.1. Ang-1 and Ang-2 Mobilize Calcium from Intracellular Calcium Stores

**Figure 2:**
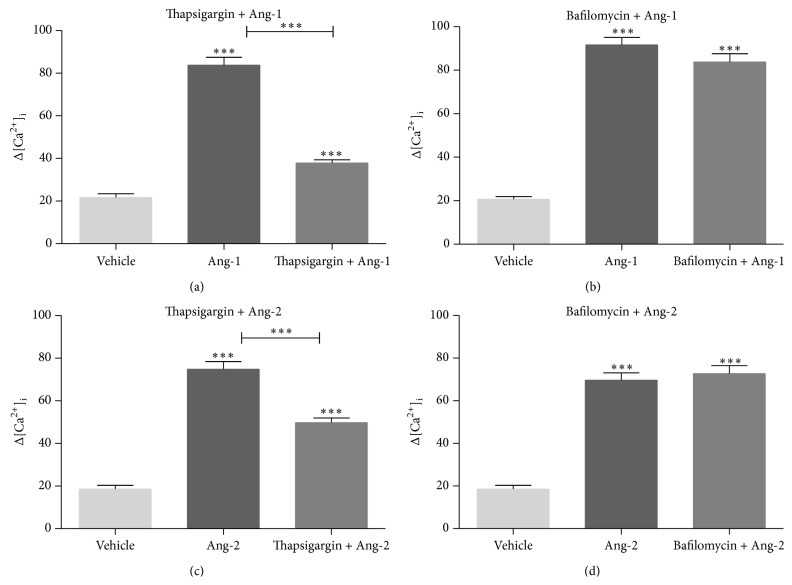
Ang-1 and Ang-2 mobilize [Ca^2+^]_i_ from ER but not from acidic stores. Live imaging in Fura-2-AM-loaded single cells. Identification of Ang-1- and Ang-2-activated [Ca^2+^]_i_ stores. Histograms represent Ca^2+^ release in cells stimulated with 100 ng/mL Ang-1 or 200 ng/mL Ang-2 after pretreatment with vehicle alone, or with 1 *μ*M thapsigargin for 15 min (a, c), or with 500 nM bafilomycin A1 for 1 h in Ca^2+^-free medium KHH (b, d). Δ on the *y*-axes = nM. Data in bar charts represent mean ± s.e.m. from three independent experiments. Cell number = 68–187. ^***^ *P* < 0.001 by Student's *t*-test.

In the present study, the involvement of Ca^2+^ in the signaling of Angs was tested and analysed to investigate in ECs if Ca^2+^ is involved in responses to Angs. We performed Ca^2+^ imaging experiments in HUVECs monolayer using microfluorimetric analysis. First, we performed dose response experiments stimulating Fura-2-AM-loaded HUVECs with Angs at different concentrations (5 ng/mL–400 ng/mL) using HBSS buffer that allows Ca^2+^ entry from the extracellular environment. The maximum increase in intracellular Ca^2+^ concentration was found to take place at the concentration of 100 ng/mL for Ang-1 and 200 ng/mL for Ang-2 (Figures 1(a) and 1(b)). These two concentrations were therefore used in all the subsequent experiments. In order to investigate the involvement of extracellular Ca^2+^ influx, we performed Ca^2+^ imaging experiments in Ang-1/Ang-2-stimulated cells using Krebs-Henseleit-HEPES buffer (KHH) and a Ca^2+^-free buffer containing the extracellular Ca^2+^ chelator EGTA (Figures 1(c1) and 1(d1)). Representative [Ca^2+^]_i_ profiles are shown in Figures 1(c2) and 1(d2). The response to Angs was found to be buffer-independent, indicating the involvement of intracellular stores. Taken together, these data show for the first time that Angs stimulation triggers intracellular Ca^2+^ signaling. The substantially equal values observed in presence or absence of extracellular Ca^2+^ prompted us to evaluate the involvement of different intracellular compartments in Angs-induced intracellular Ca^2+^ mobilization. We adopted a pharmacological approach using bafilomycin A1, which inhibits pH-dependent Ca^2+^ uptake into acidic stores by inhibition of the vacuolar-type H^+^-ATPase pump, and thapsigargin, which inhibits ER SERCA pumps [29]. Angs-induced Ca^2+^ release was significantly impaired by thapsigargin (Figures 2(a) and 2(c)) but not by bafilomycin A1 (Figures 2(b) and 2(d)), demonstrating that Ca^2+^ stores different from acidic compartments are involved in this process.

### 3.2. Both cADPR and IP_3_ Are Involved in [Ca^2+^]_i_ Increase Induced by Ang-1 and Ang-2

**Figure 3:**
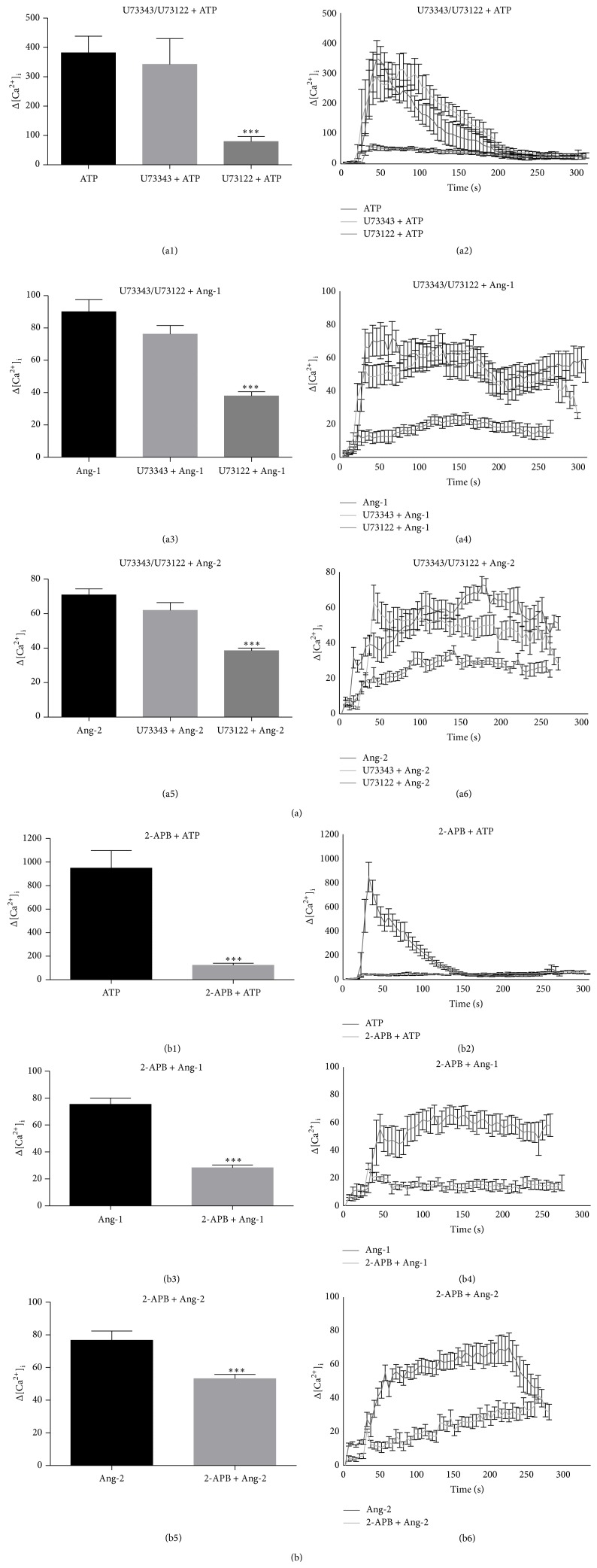
Ang-1- and Ang-2-dependent [Ca^2+^]_i_ mobilization is inhibited by antagonists of PLC and of IP_3_R. Cells pretreated for 20 min with 2 *μ*M U73122 (antagonist of PLC) or U73343 (its nonfunctional analogue) and cells pretreated for 30 min with 75 *μ*M 2-APB (selective antagonist of IP_3_ receptor) were stimulated with 10 *μ*M ATP (positive control) or Angs in Ca^2+^-free medium KHH. Statistical evaluation of Δ [Ca^2+^]_i_ in response to each agonist in the presence or absence of the indicated inhibitors (a1, a3, a5, b1, b3, and b5). Changes in [Ca^2+^]_i_ levels are shown as representative traces indicating the effect of U73122/U73343 or 2-APB on cells stimulated with ATP or Ang-1 or Ang-2 in Ca^2+^-free medium (a2, a4, a6, b2, b4, and b6). Δ on the *y*-axes = nM. Data in bar charts represent mean ± s.e.m. from three independent experiments. Cell number = 88–140. ^***^ *P* < 0.001 versus agonists by Student's *t*-test. [Ca^2+^]_i_ values in representative traces are expressed as increase with respect to basal Ca^2+^ concentrations (Δ).

Many cell stimuli act on receptors that are coupled to phospholipase C (PLC) that hydrolyses phosphatidylinositol 4,5-bisphosphate (PIP_2_) producing IP_3_, in turn recognised by receptors located on the ER resulting in the release of Ca^2+^. The spatially and temporally organized pattern of Ca^2+^ release is a remarkably versatile signaling system controlling a multitude of processes in many different cell types [34]. Another Ca^2+^ mobilizing messenger, unrelated to IP_3_, has been identified as cyclic ADP-ribose (cADPR) which activates the ryanodine receptors (RyRs) in the sarco/ER [28]. To explore the involvement of these two different second messengers responsible for Ang-1- and Ang-2-dependent intracellular Ca^2+^ mobilization from ER stores, a pharmacological approach was adopted using inhibitors specifically targeting either of them at different levels. The involvement of IP_3_ was studied through Ca^2+^ imaging experiments in cells pretreated for 20 min with 2 *μ*M U73122, the antagonist of PLC, or U73343, its nonfunctional analogue, and in cells pretreated for 30 min with 75 *μ*M 2-APB, the selective antagonist of IP_3_ receptor, and stimulated with 10 *μ*M ATP as positive control or Ang-1 or Ang-2. Pretreatment with either inhibitor significantly impaired Ang-1- and Ang-2-induced Ca^2+^ release (Figures 3(a3), 3(a5), 3(b3), and 3(b5)) and, as expected, blocked ATP-evoked Ca^2+^ mobilization, known to be dependent by IP_3_ (Figures 3(a1) and 3(b1)). Representative [Ca^2+^]_i_ traces are shown in Figures 3(a2), 3(a4), 3(a6), 3(b2), 3(b4), and 3(b6).

**Figure 4:**
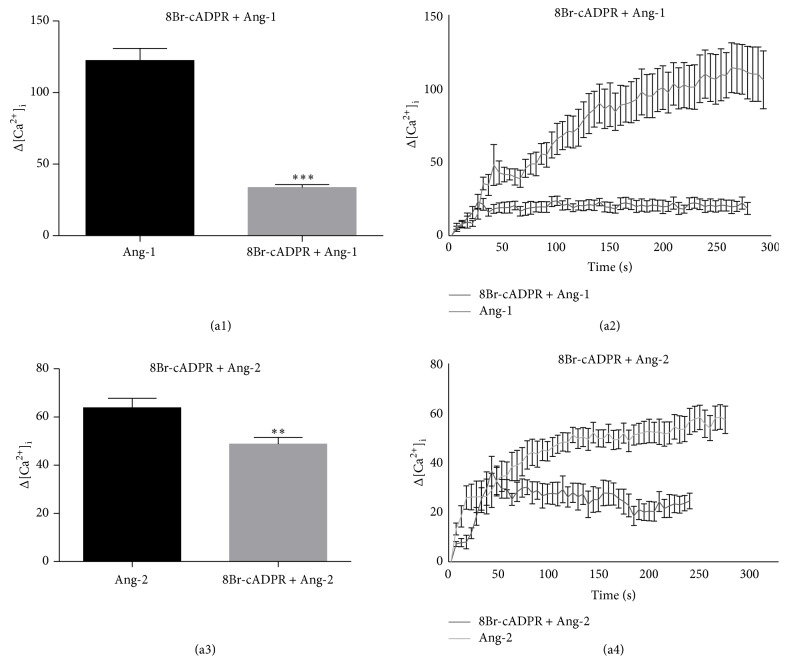
RyR inhibition abates both Ang-1- and Ang-2-mediated intracellular calcium mobilization. Cells were pretreated for 30 min with 30 *μ*M 8Br-cADPR (inactive analogue of cADPR) and then stimulated with Ang-1 or Ang-2 in Ca^2+^-free medium KHH. Histograms show changes in [Ca^2+^]_i_ levels in response to each agonist in the presence or absence of the indicated inhibitor (a1, a3). Representative traces showing the effect of 8Br-cADPR on Ang-1-stimulated (a2) and Ang-2-stimulated (a4) cells. Δ on the *y*-axes = nM. Data in bar charts represent mean ± s.e.m. of three independent experiments. Cell number = 96–132. ^**^ *P* < 0.01, ^***^ *P* < 0.001 versus agonist by Student's *t*-test. [Ca^2+^]_i_ values in representative traces are expressed as increase with respect to basal Ca^2+^ concentrations (Δ).

To identify the possible involvement of cADPR, Ca^2+^ imaging experiments were performed on cells pretreated for 30 min with 30 *μ*M 8Br-cADPR, the cell permeant antagonist of cADPR, that binds ER RyRs and then stimulated with Ang-1 or Ang-2. As shown in Figure 4, intracellular Ca^2+^ release after stimulation with either Ang was significantly reduced in cells pretreated with this inhibitor (Figures 4(a1) and 4(a3)). Representative [Ca^2+^]_i_ traces are shown in Figures 4(a2) and 4(a4). These observations demonstrate that Angs mobilize Ca^2+^ from ER stores through IP_3_ and cADPR signaling and that the involvement of either pathway is Ang-1/2 isoform specific. In particular, the unexpected partial insensitivity to thapsigargin, U73122, 2-APB, and 8Br-cADPR, observed with Ang-2 stimulation, suggests that this agonist might possibly rely upon additional Ca^2+^ storing compartments/subcompartments [35].

### 3.3. Ang-1- and Ang-2-Dependent MAPK Pathways Are Differently Regulated by IP_3_ and cADPR

**Figure 5:**
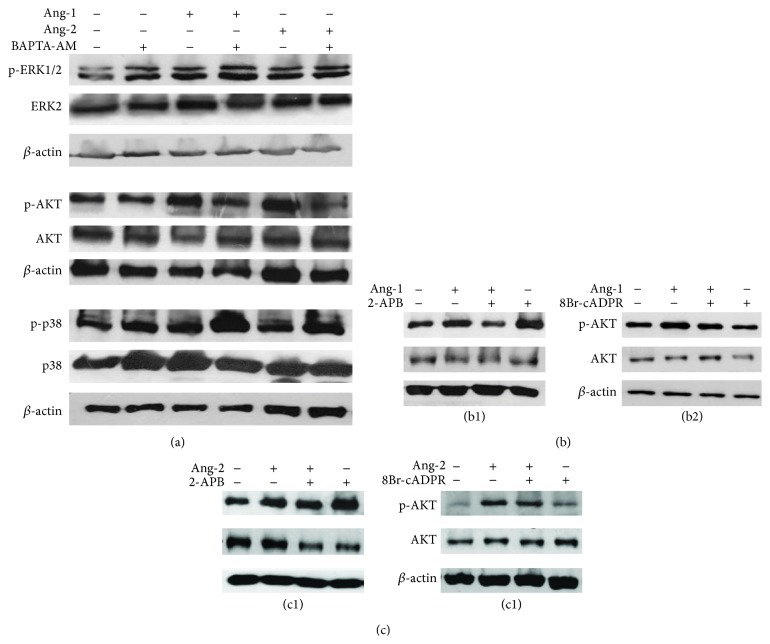
Calcium-dependent AKT and MAPK activation upon Ang-1/Ang-2 stimulation. Ang-1 and Ang-2 induce Ca^2+^-dependent AKT, ERK1/2, and p38 phosphorylation. Confluent HUVECs were pretreated or not with 20 *μ*M BAPTA-AM for 1 h and stimulated with 100 ng/mL Ang-1 or 200 ng/mL Ang-2 for 30 min. Blots of total cell lysate were probed for the phosphorylation of downstream targets AKT, ERK1/2, and p38. To ensure equal loading membranes were reprobed for the total amount of the indicated proteins and for *β*-actin. Representative blots are shown from three to five independent experiments (a). Cells pretreated or not with 75 *μ*M 2-APB or 30 *μ*M 8Br-cADPR and stimulated with either Ang-1 or Ang-2. Blots of total cell lysate were probed for the phosphorylation of downstream target AKT. To ensure equal loading membranes were reprobed for the total amount of the indicated protein and for *β*-actin. Representative blots are shown from three independent experiments (b1, b2, c1, and c2).

Ang-1/Ang-2 signaling via Tie-2 receptor is known to control specific essential steps during angiogenesis, a process involving a complex network of intracellular transduction pathways. The interaction of Ang-1 with Tie-2 in the adult vascular system is essential for ECs survival, migration, and vascular repair. It is known that in ECs Ang-1 induces phosphorylation of AKT [36], ERK1/2 [27], and p38 mitogen-activated protein kinase (MAPKs) [37]. Alitalo's group [38] demonstrated that Ang-1 and Tie-2 form distinct signaling complexes depending on the presence or absence of inter-ECs adhesion linked to the predominant phosphorylation of AKT and weaker activation of ERK in HUVECs monolayer. The intracellular phosphatidylinositol 3-kinase (PI3K)/AKT pathway is a key regulator of several cellular processes and promotes cell survival in response to various stresses such as nutrient deprivation. Among the many signaling pathways that respond to stress, the members of MAPK family are also essential for maintaining cell viability. Several studies demonstrated that Ang-1 promotes cell survival activating the MAPK and PI3K/AKT pathways [16, 19]. During the early stages of angiogenesis, neovascular sprouts are composed primarily by ECs. As they mature, microvessels acquire a coating of mural cells, which are critical for the development and maintenance of functional vasculature. In a rat aorta model [39] p38 MAPK has been shown to transduce signals critical for vascular remodeling and maturation. With the aim of identifying the involvement of Ca^2+^ in Angs-dependent signaling events, we evaluated the activation of their known protein targets ERK1/2, AKT, and p38. HUVECs were pretreated or not with the Ca^2+^ chelator BAPTA-AM at the concentration of 20 *μ*M. As shown in Figure 5(a) and Figures S1 A–F (in Supplementary Material available online at http://dx.doi.org/10.1155/2015/965271), both Angs activate the phosphorylation of AKT, ERK1/2, and p38 and the combined treatment with BAPTA-AM was found to inhibit both Ang-1- and Ang-2-dependent AKT phosphorylation, while the phosphorylation of ERK1/2 was not affected. The lack of intracellular calcium had an opposite effect on p38 phosphorylation. To further evaluate the specific contribution of the different Ca^2+^ mobilizing second messengers in AKT phosphorylation, we performed Western blot analysis of cells with two specific inhibitors, either 2-APB (for IP_3_/IP_3_R) or 8Br-cADPR (for cADPR/RyR) prior to Angs stimulation. As shown in Figures 5(b) and 5(c) and Figures S1 G–L, the phosphorylation of AKT is differentially regulated by the two Angs. Pretreatment with 2-APB specifically reduced the extent of AKT phosphorylation resulting from stimulation with Ang-1 suggesting the requirement of IP_3_-dependent Ca^2+^ release (Figures 5(b1) and 5(c1)), while cADPR/RyR signaling is significantly not involved in this response to either Ang-1 or Ang-2 (Figures 5(b2) and 5(c2)).

### 3.4. Different Ca^2+^ Signatures Regulate the Cell Migratory Response to Ang-1 and Ang-2

**Figure 6:**
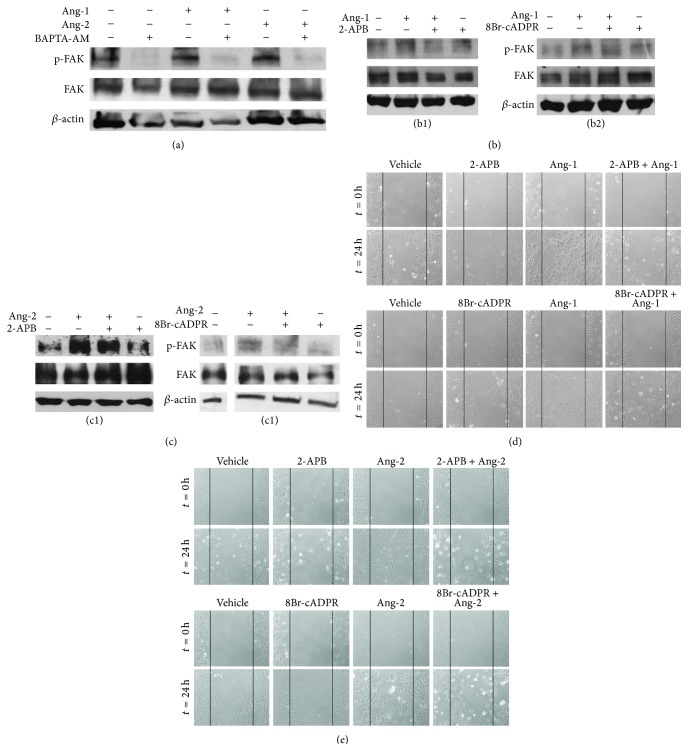
2-APB and 8Br-cADPR impair Angs-dependent cell migration on HUVECs. Ang-1 and Ang-2 induce Ca^2+^-dependent FAK phosphorylation. Confluent HUVECs were pretreated or not with 20 *μ*M BAPTA-AM for 1 h and stimulated with 100 ng/mL Ang-1 or 200 ng/mL Ang-2 for 30 min. Blots of total cell lysate were probed for the phosphorylation of downstream target FAK. To ensure equal loading membranes were reprobed for the total amount of the indicated protein and for *β*-actin (a). Cells pretreated or not with 75 *μ*M 2-APB or 30 *μ*M 8Br-cADPR and stimulated with either Ang-1 or Ang-2 (b1, b2, c1, and c2). Representative blots are shown from three to five independent experiments. Ang-1-induced EC migration is affected by treatment with the second messengers inhibitor 2-APB while Ang-2-induced cell migration appears to be both IP_3_- and cADPR-dependent. Scratch assay to evaluate the cell migration capability in the indicated experimental conditions. Wounded monolayers at the time of manual damage (*t* = 0 h upper panel) and after 24 h treatment (lower panel) with Ang-1 (d), Ang-2 (e), and inhibitors as indicated. Pictures are representative of three independent experiments.

During neovascularization and angiogenesis ECs degrade the basement membrane and migrate into the perivascular stroma in response to a gradient of angiogenic factors. Cell migration and invasion of extracellular matrix beneath the basement membrane are essential steps involving reorganization of the actin cytoskeleton. The focal adhesion kinase FAK, a member of nonreceptor tyrosine kinases, plays a key role in regulating the dynamic changes in actin cytoskeleton reorganization involved in migration and adhesion. Ang-1 induces capillary sprouting activity through nondirectional and directional migration mediated by Tie-2 but not Tie-1 receptor and induces tyrosine phosphorylation of FAK which is dependent on PI3K activity [40–43]. To identify the possible involvement of [Ca^2+^]_i_ rise in Angs-dependent activation of p-FAK, we performed Western blot analysis in cells treated or not with the Ca^2+^ chelator BAPTA-AM at the concentration of 20 *μ*M prior to stimulation with either Ang-1 or Ang-2. We observed that both Angs activate p-FAK through a Ca^2+^-dependent mechanism (Figure 6(a), Figures S2 A-B). To evaluate the contribution of the different second messengers involved, cells were pretreated with two specific inhibitors, 2-APB and 8Br-cADPR. FAK phosphorylation induced by Angs was found to depend on IP_3_ signal in cells stimulated with Ang-1 (Figures 6(b1) and 6(b2)) and strongly by both second messengers in those stimulated with Ang-2 (Figures 6(c1) and 6(c2), Figures S2 C–F). To further test the specific involvement of IP_3_ and cADPR in Ang-1- and Ang-2-induced cell motility, we performed a “scratch assay” in which confluent ECs monolayer is manually wounded along a narrow line and subsequent cell migration to reform the monolayer can be studied. The advantage of this method is that cell migration can be monitored over time, thus allowing us to estimate the rate of migratory response. Quantification is arbitrary, depending on size of wound and cell growth. In order to discriminate between migration and proliferation, flow cytometric assessment of cell proliferation with propidium iodide (PI) was performed (Figure S3). This assay showed that, in the culture conditions of the wound healing assay, ECs do not proliferate under Angs stimulation. From a qualitative point of view, the wound healing assay clearly demonstrated that the capability of ECs to move in 24 h under Ang-1 stimulation is modified by the treatment with 2-APB, while under Ang-2 stimulation cell migration is reduced by the treatment with either 8Br-cADPR or 2-APB (Figures 6(d) and 6(e)).

### 3.5. Capillary-Like Network Formation Controlled by Ang-1 Is IP_3_-Dependent

**Figure 7:**
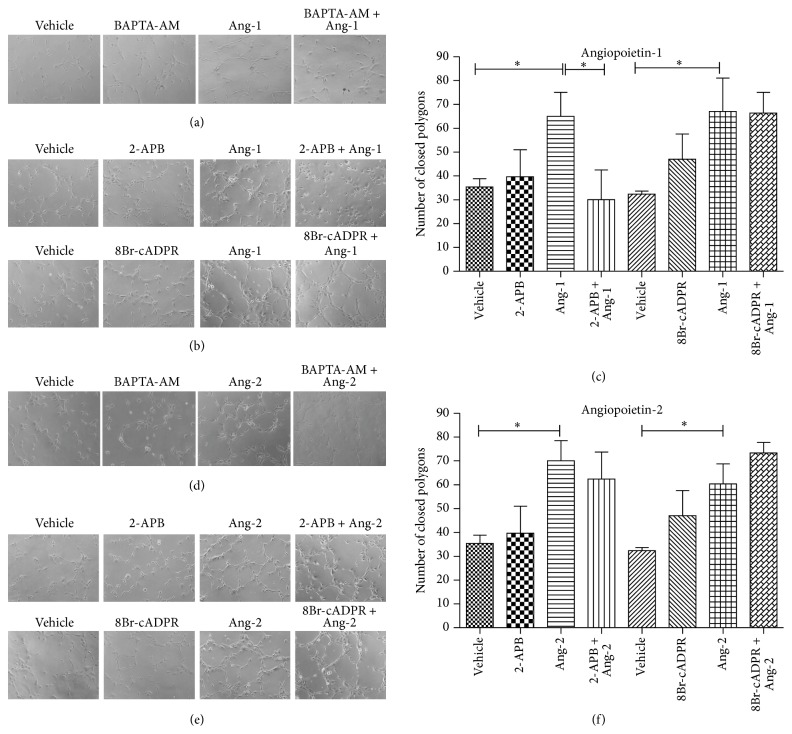
IP_3_ pathway inhibition impairs Ang-1-induced capillary-like formation* in vitro*. Cells were plated in Matrigel-coated dishes and incubated in EBM-2 + 2% FBS for 5 h in the presence or absence of Angs or/and inhibitors as indicated. Each condition was tested in triplicate. Representative images of one of three independent experiments (Ang-1: a, b; Ang-2: d, e). Quantitative evaluation of tube formation as the number of closed polygons formed in seven fields for each experimental condition (c, f). Data in bar charts represent mean ± s.e.m. of three independent experiments. ^*^ *P* < 0.05 by Student's *t*-test.

The formation of capillary-like structures* in vivo* is considered as representative of later, differentiative stages of angiogenesis and is commonly assayed to test the efficiency of compounds with pro- or antiangiogenic functions. The Angs-Tie axis is essential for vasculogenesis, normal vascular development, and angiogenesis. When ECs are plated onto a layer of gel matrix, they are stimulated to migrate and differentiate into tubular-like structures simulating the* in vivo* process [44]. Matrigel, a complex compound that mimics the extracellular matrix, containing extracellular and basement membrane proteins, is known to be the most potent matrix for tubule formation. This is usually assayed in the presence of potential modulators of angiogenesis and tubule development is observed over a 4 to 24 h period. Cord-like capillary structures are visualized as close polygons and the degree of complexity of the structure can be quantitatively evaluated counting the number of close polygons or associating a score depending on the number of ECs composing each of them. As shown in Figures 7(a) and 7(d), when plated on Matrigel matrices at high densities, HUVECs form cord-like capillary structures within a few hours, and this process is enhanced by Ang-1 and Ang-2. To evaluate the possible involvement of calcium signals in the regulation of this angiogenic process, cells were pretreated with the Ca^2+^ chelator BAPTA-AM at the concentration of 20 *μ*M. The formation of Angs-induced capillary-like structures was impaired by this inhibitor in Ang-1 stimulated samples but unaffected in cells stimulated with Ang-2, indicating that this response to Ang-2 is [Ca^2+^]_i_-independent. To further assess whether Ca^2+^ signal is dispensable for Ang-2 to induce capillary-like formation, we performed the Matrigel assay in the presence of second messengers inhibitors as shown in Figures 7(e) and 7(f) and found no evident inhibition of tube formation. Moreover, to identify by which second messenger Ang-1 controls the formation of capillary-like structures, cells were pretreated with the same second messenger inhibitors. Representative images show that this response to Ang-1 was inhibited by 2-APB pretreatment (Figure 7(b)). An approximate estimate of the efficiency of this process can be inferred by the extent of cellular network formation, whereby cells first align to form linear segments and subsequently interconnect to form closed polygonal structures. As shown in Figure 7(c), the number of closed polygons formed in cells stimulated with Ang-1 in the presence of 2-APB is significantly reduced compared with samples stimulated with Ang-1 alone, indicating the involvement of IP_3_-mediated Ca^2+^ signaling in capillary-like formation* in vitro*.

## 4. Conclusions

During the complex process of angiogenesis, a variety of growth factors and cytokines are upregulated and exert their functions through autocrine/paracrine signaling. Among these, Ang-1 and Ang-2 play important roles. Ang-1 does not stimulate ECs growth but rather promotes stabilization of vascular network and branching morphogenesis in* in vitro* and* in vivo* angiogenesis; conversely, Ang-2, a somehow context-dependent antagonist of Ang-1, promotes vessels destabilization favouring pericytes detachment [45]. In the present study, we identified a novel Ca^2+^-dependent machinery activated by Angs which controls angiogenic processes, including migration and the ability to induce capillary-like structures* in vitro*. Our data suggest that Ca^2+^ signaling might be envisaged as a possible “signaling hub” for Angs and other angiogenic factors. Given the therapeutic potential of angiogenesis-blocking therapies based on the inhibition of specific signaling pathways, this concept could open the way to novel experimental approaches in this field.

## Supplementary Material

Densitometric Analysis of Western Blots: Densitometry was performed on scanned immunoblot images using the ImageJ analysis tool to obtain the intensity for each experimental band calculated by normalizing to the correspond control β-actin band.
