# Supplementary material for: Regulation of Angiogenic Functions by Angiopoietins through Calcium-Dependent Signaling Pathways
Source: Biomed Res Int. 2015 Jun 4;2015:965271. doi: 10.1155/2015/965271 (PMC4471310; doi:10.1155/2015/965271)
Supplement: Supplementary file 1 — Densitometric Analysis of Western Blots: Densitometry was performed on scanned immunoblot images using the ImageJ analysis tool to obtain the intensity for each experimental band calculated by normalizing to the correspond control β-actin band. [file 965271.f1.pdf]

Supplementary Materials and Methods

Flow cytometric assessment of cell proliferation

Cell cultured in EGM-2 as monolayer were stimulated with 100ng/ml Ang-1 or 200ng/ml Ang-2 for 24h. Cells were harvested by trypsin/EDTA (Sigma), rinsed with PBS+1%BSA (Sigma) and incubated with 2µg/ml propidium iodide (PI, Sigma) plus 0,1 U/L of RNAse. Stimulated and unstimulated cells were then counted in a Coulter Epics XL flow cytometer (Beckman Coulter) and data were analyzed using FCS3 express Software (De Novo Software).

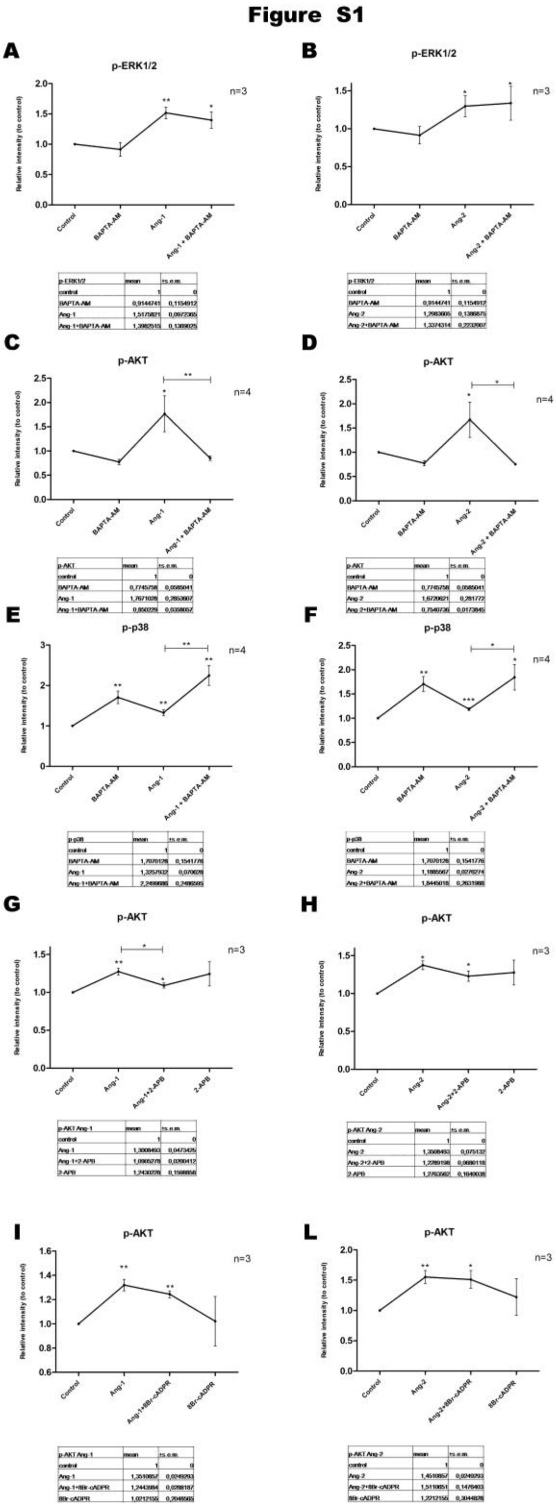

p-ERK1/2	mean	±s.e.m.
control	1	0
BAPTA-AM	0,9144741	0,1154912
Ang-1	1,5175821	0,0972365
Ang-1+BAPTA-AM	1,3982515	0,1369025

p-ERK1/2	mean	±s.e.m.
control	1	0
BAPTA-AM	0,9144741	0,1154912
Ang-2	1,2983605	0,1386875
Ang-2+BAPTA-AM	1,3374314	0,2232907

p-AKT	mean	±s.e.m.
control	1	0
BAPTA-AM	0,7745758	0,0585041
Ang-1	1,7671026	0,2853807
Ang-1+BAPTA-AM	0,850229	0,0358057

p-AKT	mean	±s.e.m.
control	1	0
BAPTA-AM	0,7745758	0,0585041
Ang-2	1,6720621	0,281772
Ang-2+BAPTA-AM	0,7540736	0,0173845

p-p38	mean	±s.e.m.
control	1	0
BAPTA-AM	1,7070126	0,1541776
Ang-1	1,3257932	0,070628
Ang-1+BAPTA-AM	2,2499686	0,2499565

p-p38	mean	±s.e.m.
control	1	0
BAPTA-AM	1,7070126	0,1541776
Ang-2	1,1885567	0,0276274
Ang-2+BAPTA-AM	1,8445018	0,2631988

p-AKT Ang-1	mean	±s.e.m.
control	1	0
Ang-1	1,3008493	0,0473425
Ang-1+2-APB	1,0905278	0,0290412
2-APB	1,2430228	0,1568858

p-AKT Ang-2	mean	±s.e.m.
control	1	0
Ang-2	1,3508493	0,075132
Ang-2+2-APB	1,2289198	0,0680118
2-APB	1,2763562	0,1640038

p-AKT Ang-1	mean	±s.e.m.
control	1	0
Ang-1	1,3510857	0,0249293
Ang-1+8Br-cADPR	1,2443984	0,0288187
8Br-cADPR	1,0212155	0,2048565

p-AKT Ang-2	mean	±s.e.m.
control	1	0
Ang-2	1,4510857	0,0249293
Ang-2+8Br-cADPR	1,5110851	0,1476403
8Br-cADPR	1,2212155	0,3044828

<u>Figure S1</u>: Quantification of calcium-dependent AKT and MAPK activation upon Ang-1/Ang-2 stimulation

Densitometric quantification of Western blots showing levels of p-ERK1/2/β actin, p-AKT/ β actin, p-p38/ β actin versus control (vehicle, set as 1) in the culture conditions detailed in the legend to Fig.5. Data are expressed as mean ± s.e.m. from at least three independent experiments. Student's *t-test* was used for statistical comparison between means. *p<0.05, **p<0.01, ***p<0.001.

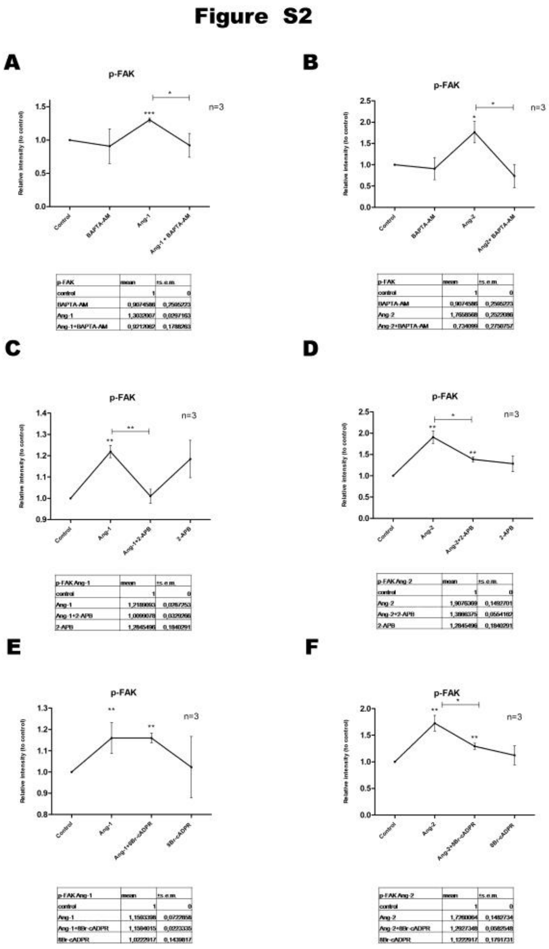

<u>Figure S2</u>: Quantification of calcium-dependent FAK activation upon Ang-1 and Ang-2 stimulation

Densitometric quantification of Western blots showing levels of p-FAK/β actin versus control (vehicle, set as 1) in the culture conditions detailed in the legend to Fig 6 A,B1-C2. Data are expressed as mean ± s.e.m. from at least three independent experiments. Student's *t-test* was used for statistical comparison between means. *p<0.05, **p<0.01, ***p<0.001.

Figure S3

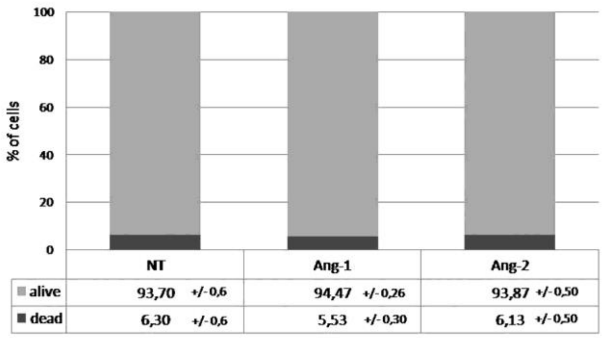

	NT	Ang-1	Ang-2
alive	93,70 +/-0,6	94,47 +/-0,26	93,87 +/-0,50
dead	6,30 +/-0,6	5,53 +/-0,30	6,13 +/-0,50

<u>Figure S3:</u> Angs treatment fails to stimulate cell proliferation

The proliferative response of HUVECs to stimulation with 100ng/ml Ang-1 or 200ng/ml Ang-2 for 24 h, was tested by flow cytometry using propidium iodide. The percentage of live and dead cells in stimulated and control (NT) conditions are shown. Values in table represent the mean ±s.e.m. from three independent experiments.
